# Comment on “Advanced Hepatic Fibrosis in Fatty Liver Disease Linked to Hyperplastic Colonic Polyp”

**DOI:** 10.1155/2017/4083272

**Published:** 2017-12-10

**Authors:** Davide Giuseppe Ribaldone, Giorgio Maria Saracco, Rinaldo Pellicano

**Affiliations:** ^1^Department of Medical Sciences, University of Torino, 10126 Torino, Italy; ^2^Department of Gastroenterology, Molinette Hospital, University of Turin, Turin, Italy

In a recent retrospective, cohort, observational study conducted at Division of Internal Medicine of the Holy Family Hospital, Nazareth, Israel, between April 2013 and April 2015 on patients who underwent screening colonoscopy, the authors concluded that for the first time an association between biopsy-proven nonalcoholic steatohepatitis (NASH) and the burden of hyperplastic polyp (HP) was shown [1]. The same group has published in Israel Medical Association Journal on the association between nonalcoholic fatty liver disease (NAFLD) and hyperplastic polyp [2].

Apart from the fact that the authors should explain the differences between these two publications [1, 2], we would like to highlight some issues.

In the first part of the paper, Mahamit et al. reported that other risk factors (compared to lifestyle and dietary risk factors) for development of HP include alcohol consumption, cigarette smoking, obesity, metabolic syndrome, and fiber intake: these are not “other” risk factors but are examples of lifestyle and dietary risk factors.

Moreover, the authors remind us that metabolic syndrome is a risk for HP and nonalcoholic fatty liver disease (NAFLD). To appropriately assess if NASH is a risk factor in the development of HP, a control population without NASH should be chosen. In this study, the control population does not statistically differ from the cases (biopsy-proven NASH) in terms of prevalence of metabolic syndrome, diabetes mellitus, hypertension, and hyperlipidemia, which are NASH risk factors [3]. Thus, although the authors reported that the controls were not affected by NASH (whose definitive diagnosis requires a liver biopsy [4]), more information on the assessment of liver status is required to minimize the risk of biases and to establish the real prevalence of HP (i.e., a familiar history positive for polyps and use of nonsteroidal anti-inflammatory drugs) in cases and controls [1].

Another criticism arises from the consideration that not all endoscopists report routinely hyperplastic-appearing diminutive rectosigmoid polyps. This is supported by the fact that current colonoscopic practice does not systematically remove all diminutive rectosigmoid lesions that appear hyperplastic and supports the “do not resect” paradigm proposed by the American Society for Gastrointestinal Endoscopy [5]. Only colonoscopies performed in a study setting with the endoscopists alerted to report all HP could give their real prevalence.

In conclusion, prospective studies are required to confirm if NASH is a risk factor for HP.
